# Supplementary material for: Regulation of Myc transcription by an enhancer cluster dedicated to pluripotency and early embryonic expression
Source: Nat Commun. 2024 May 10;15:3931. doi: 10.1038/s41467-024-48258-5 (PMC11087473; doi:10.1038/s41467-024-48258-5)

Supplementary Figures and Tables

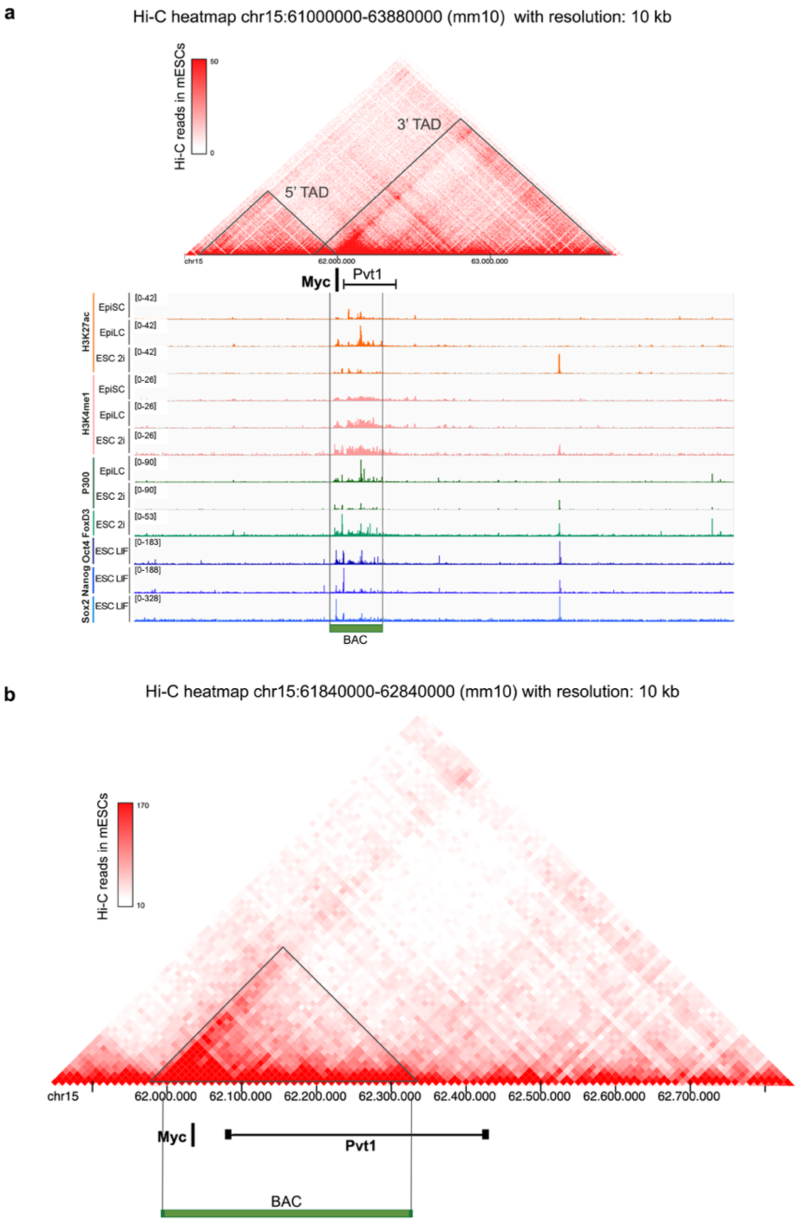

Supplementary Figure 1. Epigenetic modifications and Hi-C heatmap of the genomic region surrounding *Myc* locus in mouse ESCs. a, The 3Mb region around *Myc*, showing 2 major TADs upstream and downstream the *Myc* transcription unit. Hi-C reads in mESC from[76] are represented as a heatmap. The intensity of the red color correlates with the frequency of interactions. Scaled representation of the *Myc* 3Mb gene desert in the genome and in BAC RP24-78D24 (green box). Below, H3K27ac (orange) and H3K4me1 (pink) distribution in EpiSCs (primed pluripotency), EpiLCs (formative pluripotency) and ESCs 2i (pro-naïve pluripotency). P300 (dark green) binding in EpiLCs and naive ESCs. FOXD3 (light green), OCT4, NANOG and SOX2 (blue) binding in ESCs. Source of the data is the same as in Figure 1. **b,** 1Mb region around *Myc* of the downstream region showing a sub-TAD.

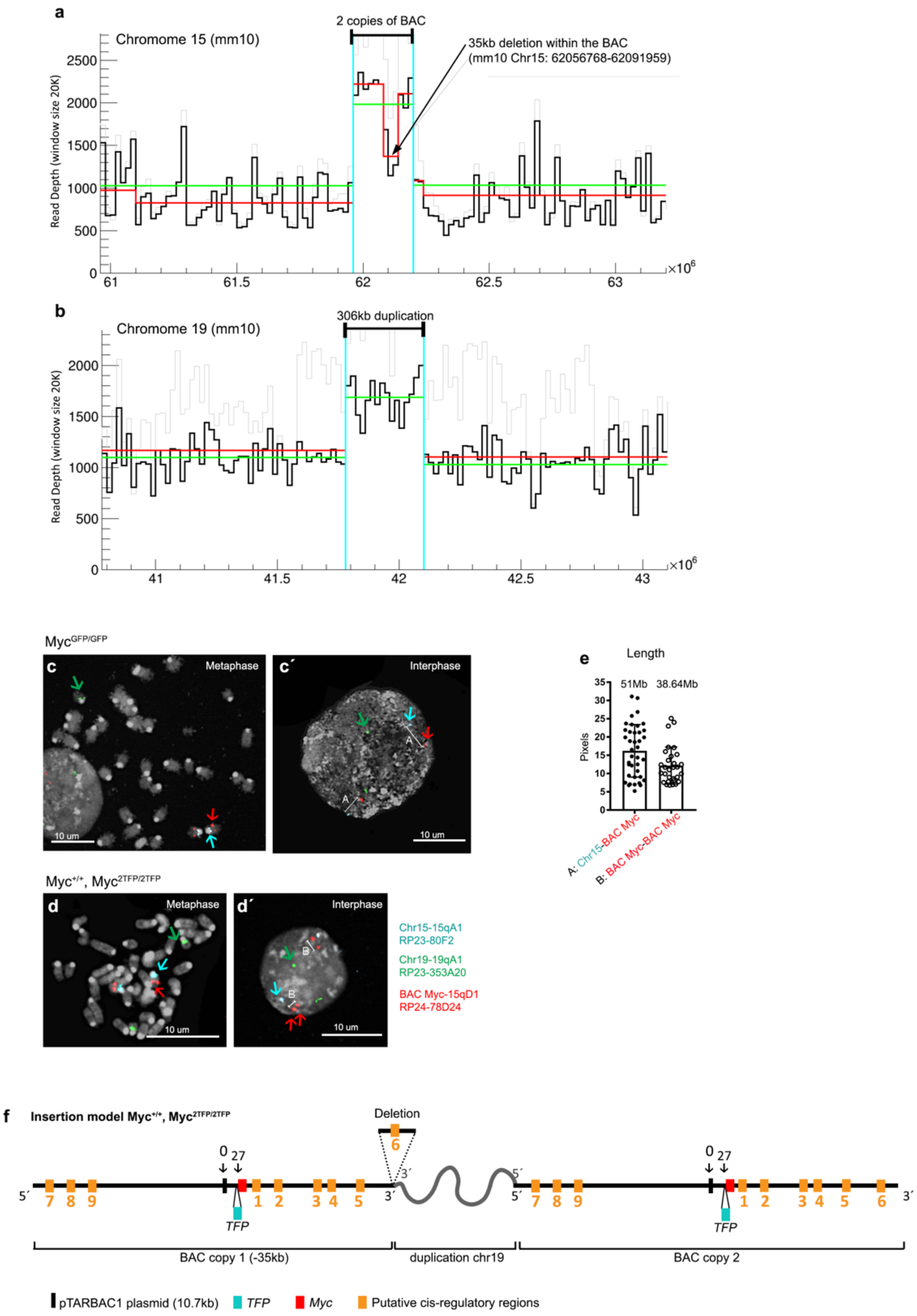

Supplementary Figure 2. Characterization of the BAC RP24-78D24 transgenic insertion. **a-b,** Graph presenting a 20Kb sliding window showing copy number detection from genomic DNA deep sequencing of mice carrying the transgene BAC transgene insertion in heterozygosis. The red line represents the read depth compared to the average in the surrounding genome. The green line represents the read depth compared to the average of the entire genome. **a,** Chromosome 15: the BAC sequences (delimited by blue lines) are detected in double amount than the surrounding sequences, indicating the detection of the 2 endogenous alleles, and 2 extra-copies from the single

insertion of the BAC. A 1-copy deletion of ~35kb within the BAC can be observed. **b,** Chromosome 19: hybrid reads between the BAC DNA and chromosome 19 DNA were detected. The region between the two breakpoints identified by the hybrid sequences was detected in 3 copies (delimited by blue lines) which suggest that the insertion site of the BAC involved the duplication of this region. **c-d,** FISH hybridization in *Myc*$^{GFP/GFP}$ (**c,c´**) and *Myc*$^{+/+}$,*Myc*$^{2TFP/2TFP}$ (**d,d´**) cells. 19qA1 region of the chr19 is tagged by a green probe (green arrows). A control 15qA1 region of chr15 is tagged by a blue probe (blue arrows). The BAC, in the 15qD1 region is labelled by a red probe (red arrows). Scale bars=10 microns. In the metaphasic nuclei (**c, d**), the red and the blue probes co-localize in chromosome 15. In interphasic nuclei (**c´, d´**), there are two signals of the red probe in *Myc*$^{GFP/GFP}$ cells, and four red signals in *Myc*$^{+/+}$, *Myc*$^{2TFP/2TFP}$ cells. **e,** Estimation of the distance between the Myc locus and the BAC insertion by comparing quantification of the distance *Myc*-Control Probe (A distance) versus Myc-BAC (B distance). From the known distance between 15qA1 and *Myc* (51Mb), the distance between *Myc* and the BAC insertion is estimated at 38.64 Mb. **f,** Insertion model of the TFP-Myc BAC into ectopic site in chromosome 15, carrying a fragment from chromosome 19. Two copies of BAC were inserted, one copy with a 35Kb deletion. Source data for all graphs are available from the Source Data file and raw data from Figshare (see Data Availability section).

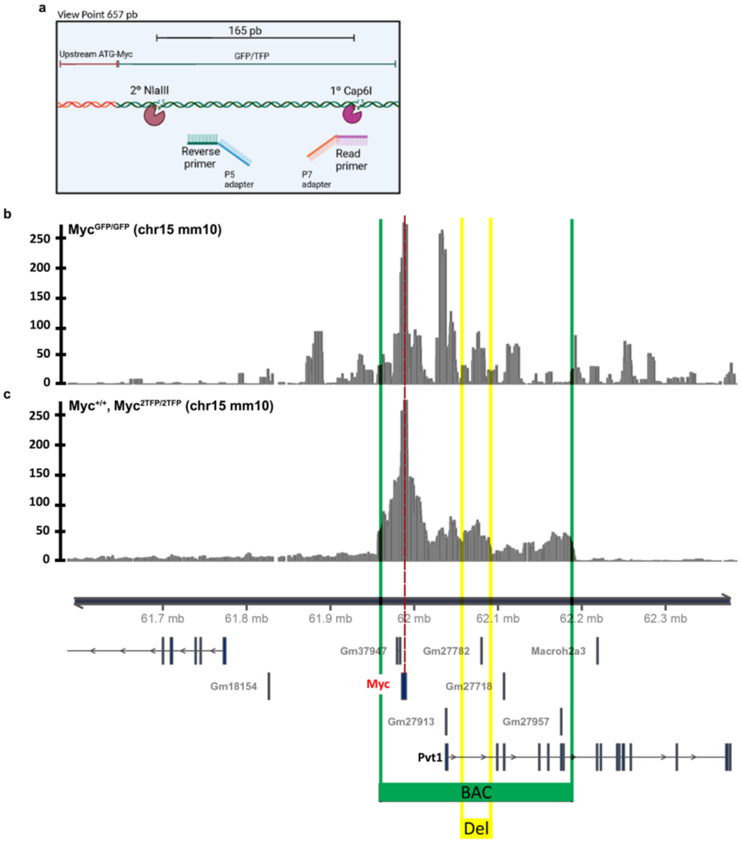

Supplementary Figure 3. 4C analysis in *Myc*$^{GFP/GFP}$ and *Myc*$^{+/+}$, *Myc*$^{2TFP/2TFP}$ ESCs. a, Viewpoint design. TFP or GFP was used as viewpoint, which is upstream and in frame with *Myc* coding sequence. The two enzymes and primers used for generating the libraries are represented. Primers are listed in Supplementary Table 2. **b-c,** Reference genes such as *Myc* (red dashed line) and *Pvt1* are represented below. The BAC region is delimited by green lines and the 35Kb deletion in the BAC transgene (Del) is delimited by yellow lines. **b,** *GFP-Myc* interactions. **c,** *TFP-Myc* interactions.

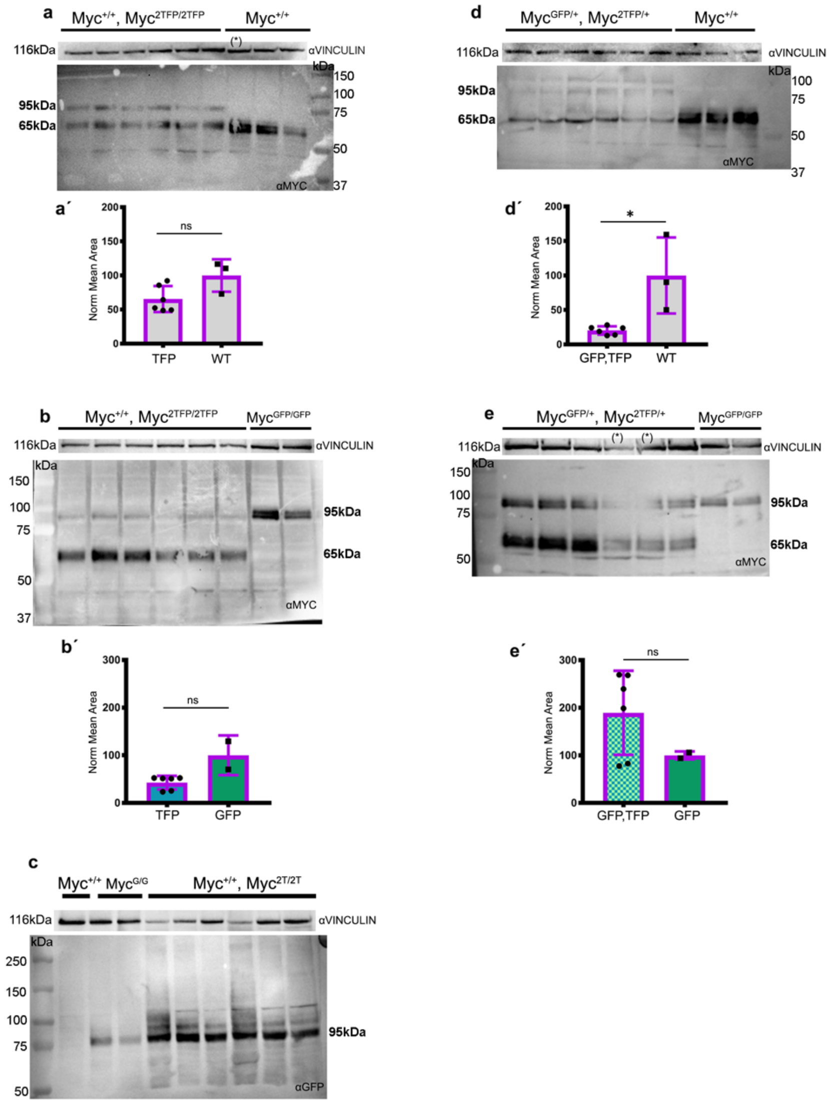

Supplementary Figure 4. Characterization of the TFP-Myc fusion protein expression. a-c, Western blot of $Myc^{+/+}$, $Myc^{2TFP/2TFP}$ (TFP) ESCs lysates. Anti-vinculin is shown as loading control. **a´,** Graphs show the abundance of WT MYC protein (65kDa) produced by TFP cells and $Myc^{+/+}$ cells (WT). **b´,** Graph shows the abundance of FP-MYC fusion protein (95kDa) produced by TFP cells and by $Myc^{GFP/GFP}$ cells (GFP). **c,** anti-GFP antibody in WT, GFP, and TFP cells, FP-MYC fusion protein is absent in WT cells, and GFP and TFP-fusion proteins had the same size (95kDa). **d-e,** Western blot of $Myc^{GFP/+}$, $Myc^{+/2TFP}$ (GFP,TFP) ESCs lysates. **d´,** Graph shows the abundance of the MYC protein produced by GFP;TFP and WT cells. **e´,** Graph shows the abundance of FP-MYC fusion protein produced by GFP;TFP cells and GFP cells. **a'- e',** Mann Whitney test; ns: P-value >0,05; *: P-value=0.0238; All quantifications were normalized to vinculin and to the control quantity of WT or FP-fusion proteins. Each dot represents an independent Western-Blot lane analysis. bars indicate the Mean and error bars, the Standard Deviation. n= 6 and 3 in **a'** and **d'** and 6 and 2 in **b'** and **e'**. The images shown here are representative examples of the different experiments quantified. (*) aberrant bands were not used for quantifications. Source data for all graphs are available from the Source Data file and raw data from Figshare (see Data Availability section).

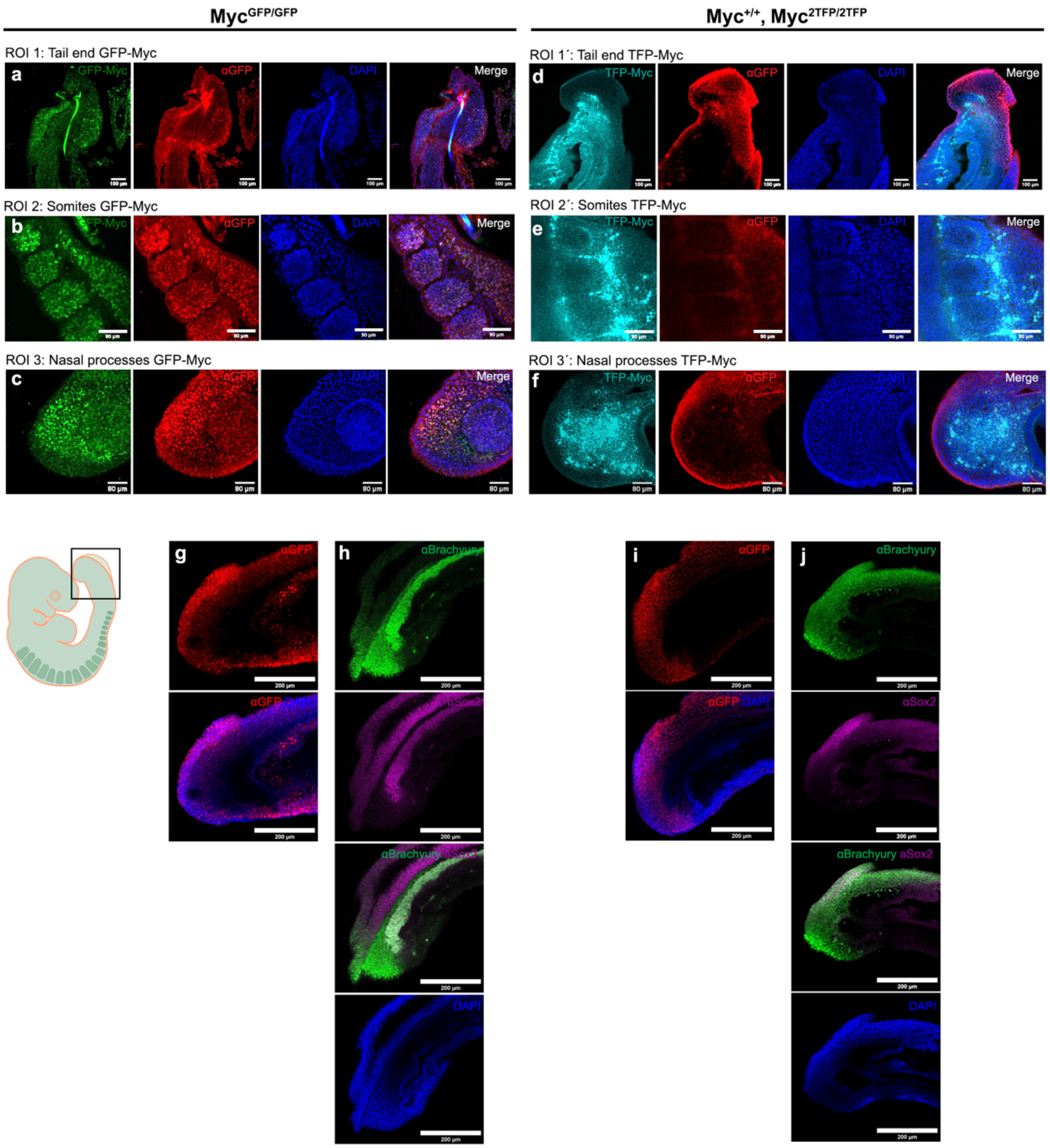

Supplementary Figure 5. Comparative *GFP-Myc* and *TFP-Myc* expression in E9.5 embryos. **a-c,** Confocal images show the posterior embryonic bud (**a**), the newly formed somites (**b**) and the nasal processes (**c**) of E9.5 $Myc^{GFP/GFP}$ embryos with detection of native GFP-MYC signal, immunostaining against GFP and DAPI. N = 6 GFP-MYC embryos. **d-f,** similar images as in **a-c** for a $Myc^{+/+}$, $Myc^{2TFP/2TFP}$ embryo. **a,d,** Scale bar= 100 microns. N = 10 TFP-MYC embryos. **b,e,** Scale bar= 60 microns. **c,f,** Scale bar= 80 microns. **g-j,** Confocal images show optical transversal sections of the posterior embryonic bud in $Myc^{GFP/GFP}$ (**g**,**h**) and $Myc^{+/+}$, $Myc^{2TFP/2TFP}$ (**I**, **j**) E9.5 embryos. Scale bar= 200 microns. FP-MYC is detected by antiGFP immunostaining (**g**, **i**) and NMPs are detected by double staining with anti-Brachyury and anti-SOX2 antobodies (**h**, **j**).

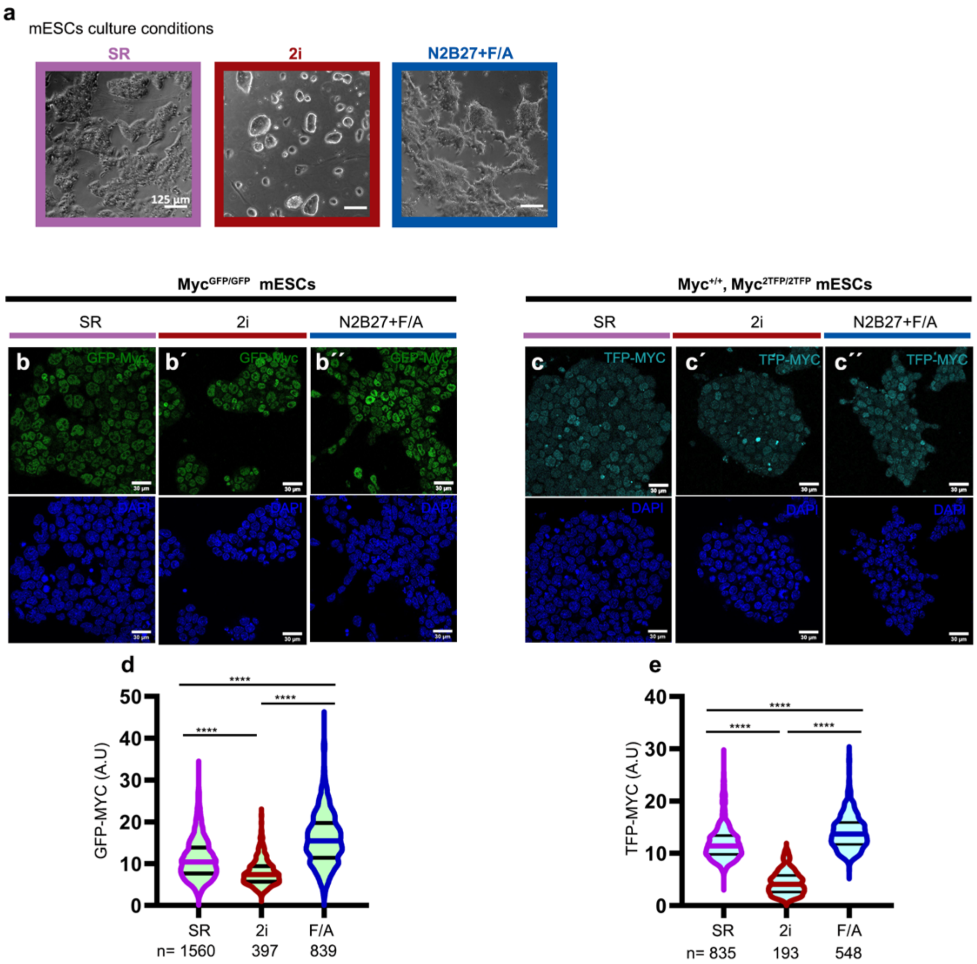

Supplementary Figure 6. *TFP-Myc* expression analysis by mESC cultured in SR+LIF, 2i and 2 days-F/A conditions. a, Brightfield images of mESCs in the three culture conditions used in this work: SR+LIF (purple), in 2i (red), and in 2 days-F/A (blue). Scale bar= 125 microns. **b-c,** Confocal images show GFP-MYC or TFP-MYC endogenous signals and DAPI in $Myc^{GFP/GFP}$ (**b-b´´**) (N=2 clones) and $Myc^{+/+}$, $Myc^{2TFP/2TFP}$ (**c-c´´**) (N=2 clones) in the three culture conditions. Scale bar= 30 microns. **d, e,** Violin plots show median and quartiles of GFP-MYC or TFP-MYC endogenous signals of $Myc^{GFP/GFP}$ (**d**) and $Myc^{+/+}$, $Myc^{2TFP/2TFP}$ (**e**) mESCs in the 3 culture conditions indicated. The number of cells quantified (n) is shown. Mann Whitney test; ns: P-value >0,05, ****: P-value<0,0001. Source data for all graphs are available from the Source Data file and raw data from Figshare (see Data Availability section).

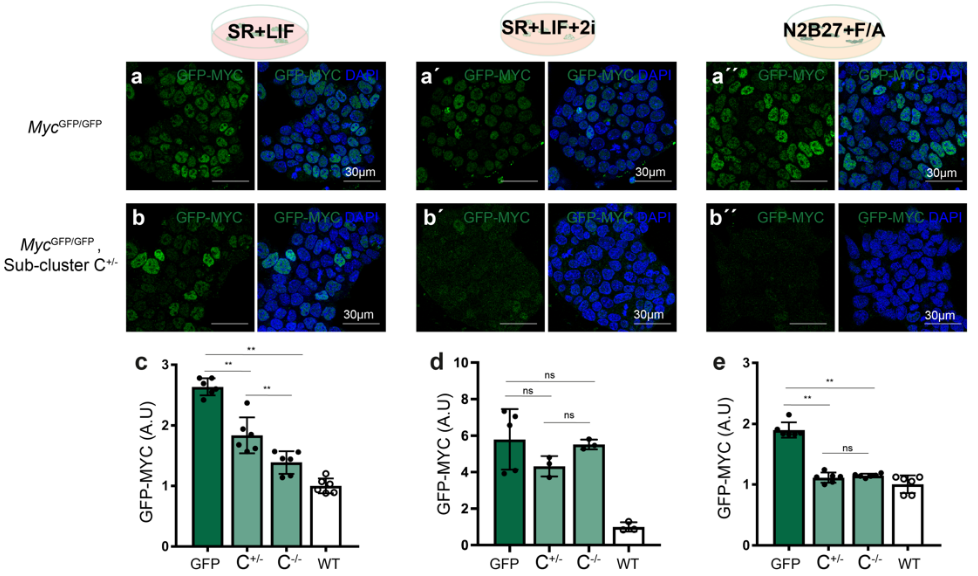

f

Sub-cluster	Putative region	Putative enhancer	position	size (bp)	cCRE ENCODE	Position	Score	Size (bp)	H3K27ac max-Z	Cell type (High H3K27ac)
c	7	7_1	chr15:62,085,072-62,088,281	3211	EM10E0600677	chr15:62085273-62085583	182	311	2.39	129 ES-E14 B10.H-2aH-4bp/Wts CH12.LX
					EM10E0600679	chr15:62087830-62088156	242	327	2.68	
		7_2	chr15:62,089,322-62,092,601	3281	EM10E0600682	chr15:62091244-62091460	219	217	3.15	
					EM10E0600683	chr15:62091806-62092032	225	227	3.69	
		7_3	chr15:62,092,596-62,095,870	3276	EM10E0600685	chr15:62092738-62093087	451	350	3.29	
					EM10E0600689	chr15:62096850-62097138	186	289	2.58	
		7_4	chr15:62,096,720-62,099,694	2976	EM10E0600690	chr15:62098817-62099164	289	348	2.63	
					EM10E0600691	chr15:62099282-62099619	338	338	2.87	

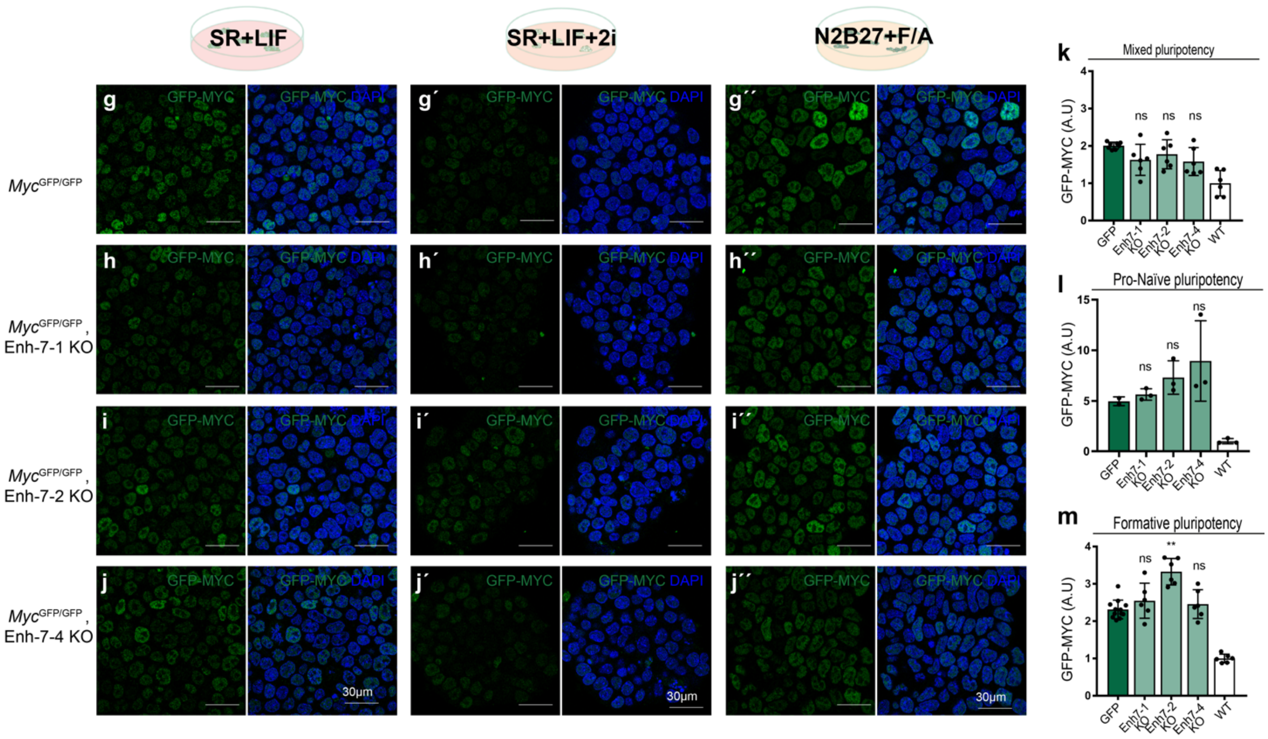

Supplementary Figure 7. *GFP-Myc* expression analysis in ESC with heterozygous deletion of Sub-cluster C and homozygous deletion of putative enhancers 7-1, 7-2 and 7-4. a-b, Confocal images show GFP-MYC endogenous fluorescence and DAPI in control $Myc^{GFP/GFP}$ cells (**a-a´´**) and cells with heterozygous deletion of sub-cluster C (**b-b´´**) in SR+LIF, SR+2i and 2 days-N2B27+F/A culture conditions. Scale bar= 30 microns. **c-e,** Graph shows normalized median intensity of sub-cluster C deletion in heterozygosity or homozygosity and comparison to GFP-Myc and Control ES cells. 3 independent clones with two biological replicates each were measured in **c** and **e**. 5 GFP-Myc independent clones and 3 independent clones for the rest of genotypes were measured in **d**. GFP-MYC endogenous levels were analyzed by FACs in SR+LIF (**c**) and 2 days-F/A (**e**) conditions. In SR+2i condition (**d**) GFP-MYC levels were analyzed in confocal images. The number of cells quantified per clone/biological replicate is available from the source data in Figshare. The bars show the Median and the error bars show the Standard Deviation. Mann Whitney test; ns: P-value >0,05; **: P-value=0.0022 except for $C^{+/-}$ vs $C^{-/-}$ comparison in which it was 0.0043. **f,** Table shows the genomic coordinates of putative enhancers and cCRE included in sub-cluster C. Name, position, size score, max-z and cell type are indicated. **g-j,** Confocal images show GFP-MYC endogenous fluorescence and DAPI in control $Myc^{GFP/GFP}$ cells (**g-g´´**) and cells with a homozygous deletion of enhancer 7-1 (**h-h´´**), enhancer-7-2 (**i-i´´**) and enhancer-7-4 (**j-j´´**) cultured in 3 conditions. Scale bar= 30 microns. **k-m,** Bars show normalized median intensity and error bars show Standard Deviation. 3 independent clones with two biological replicates each were measured in **k** and **m**, except for the GFP-MYC condition, in which 5 clones were used. 2 GFP-Myc independent clones and 3 independent clones for the rest of genotypes were measured in **d**. GFP-MYC endogenous levels were analyzed by FACs in SR+LIF (**k**) and 2 days-F/A (**l**) conditions. In SR+2i condition (**m**) GFP-MYC levels were analyzed by in confocal images. The number of cells quantified per clone/biological replicate is available from the source data in Figshare. One-way ANOVA with Dunnett's correction; ns: P-value >0,05; **: P-value=0,0011. Source data for all graphs are available from the Source Data file and raw data from Figshare (see Data Availability section).

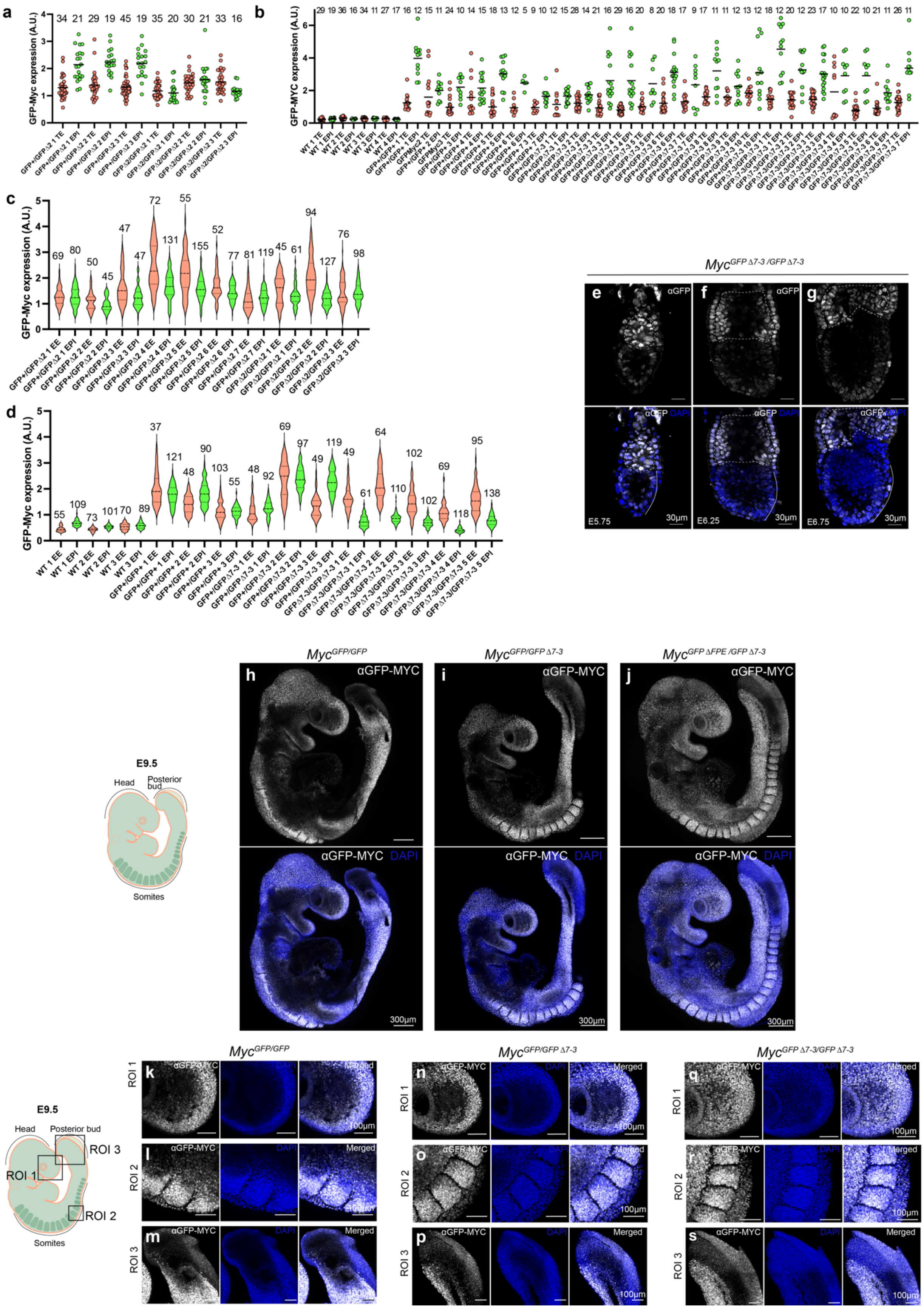
a
b
GFP-Myc expression (A.U.)
GFP-MYC expression (A.U.)
c
d
$Myc^{GFP\,\Delta7\text{-}3\,/GFP\,\Delta7\text{-}3}$
e
f
g
αGFP
αGFP DAPI
E5.75
E6.25
E6.75
30µm
$Myc^{GFP/GFP}$
$Myc^{GFP/GFP\,\Delta7\text{-}3}$
$Myc^{GFP\,\Delta FPE\,/GFP\,\Delta7\text{-}3}$
h
i
j
αGFP-MYC
αGFP-MYC DAPI
300µm
E9.5
Head
Posterior bud
Somites
ROI 1
ROI 2
ROI 3
k
l
m
n
o
p
q
r
s
Merged
100µm

Supplementary Figure 8. Analysis of the requirement for Enhancers 2 and 7-3 activity from E3.5 to E9.5. a, Dot plot showing GFP-MYC expression levels in trophectoderm (TE) and epiblast cells (EPI) of $Myc^{GFP/GFP\Delta 2}$ and $Myc^{GFP\Delta 2/GFP\Delta 2}$ blastocysts. **b**, Dot plot showing GFP-MYC expression levels in trophectoderm (TE) and epiblast cells (EPI) of wild type, $Myc^{GFP/GFP}$, $Myc^{GFP/GFP\Delta 7\text{-}3}$ and $Myc^{GFP\Delta 7\text{-}3/GFP\Delta 7\text{-}3}$ blastocysts. In **a**, **b**, each column represents an individual embryo and each dot represents the intensity of single cells, with the number of cells scored indicated above. **c,** Violin plot showing GFP-MYC expression levels in extra-embryonic ectoderm (EE) and epiblast cells (EPI) of $Myc^{GFP/GFP\Delta 2}$ and $Myc^{GFP\Delta 2/GFP\Delta 2}$ E6.5 embryos. **d**, Violin plot showing GFP-MYC expression levels in extra-embryonic ectoderm (EE) and epiblast cells (EPI) of wild type, $Myc^{GFP/GFP}$, $Myc^{GFP/GFP\Delta 7\text{-}3}$ and $Myc^{GFP\Delta 7\text{-}3/GFP\Delta 7\text{-}3}$ E6.5 embryos. In **c**, **d**, each column represents an individual embryo, and the plots represent the intensity values of single cells, with the number of cells scored indicated above each column. Violin plots show median and quartiles. **e-g,** Confocal images show anti-GFP immunofluorescence and DAPI in E5.75, E6.25 and E6.75 $Myc^{GFP\Delta 7\text{-}3/GFP\Delta 7\text{-}3}$ embryos. Scale bar= 30 microns. **h-j**, Confocal images with Maximum Intensity Z-projection show immunostaining against GFP and DAPI in $Myc^{GFP/GFP}$ (h, N=4), $Myc^{GFP/GFP\Delta 7\text{-}3}$ (i, N=5) and $Myc^{GFP\Delta 7\text{-}3/GFP\Delta 7\text{-}3}$ (j, N=6) E9.5 embryos. Scale bar= 300 microns. **k-i,** Confocal images show antiGFP immunofluorescence and DAPI in E9.5 embryos. Scale bar= 100 microns. **k-m,** Nasal process (**k**), somites (**l**) and posterior embryonic bud (**m**) regions of a $Myc^{GFP/GFP}$ embryo. **n-p,** Nasal process (**n**), somites (**o**) and posterior embryonic bud (**p**) regions of a $Myc^{GFP/GFP}$ embryo. **q-s,** Nasal process (**q**), somites (**r**) and posterior embryonic bud (**s**) regions of a $Myc^{GFP\Delta 7\text{-}3\ /GFP\Delta 7\text{-}3}$ embryo. Source data for all graphs are available from the Source Data file and raw data from Figshare (see Data Availability section).

a

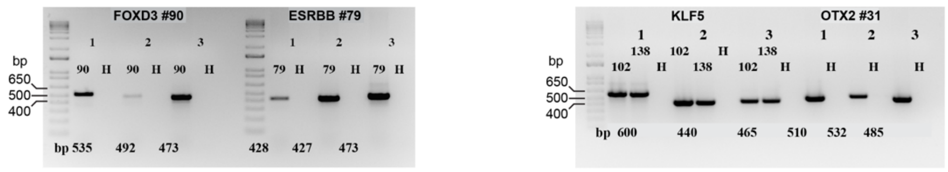

b

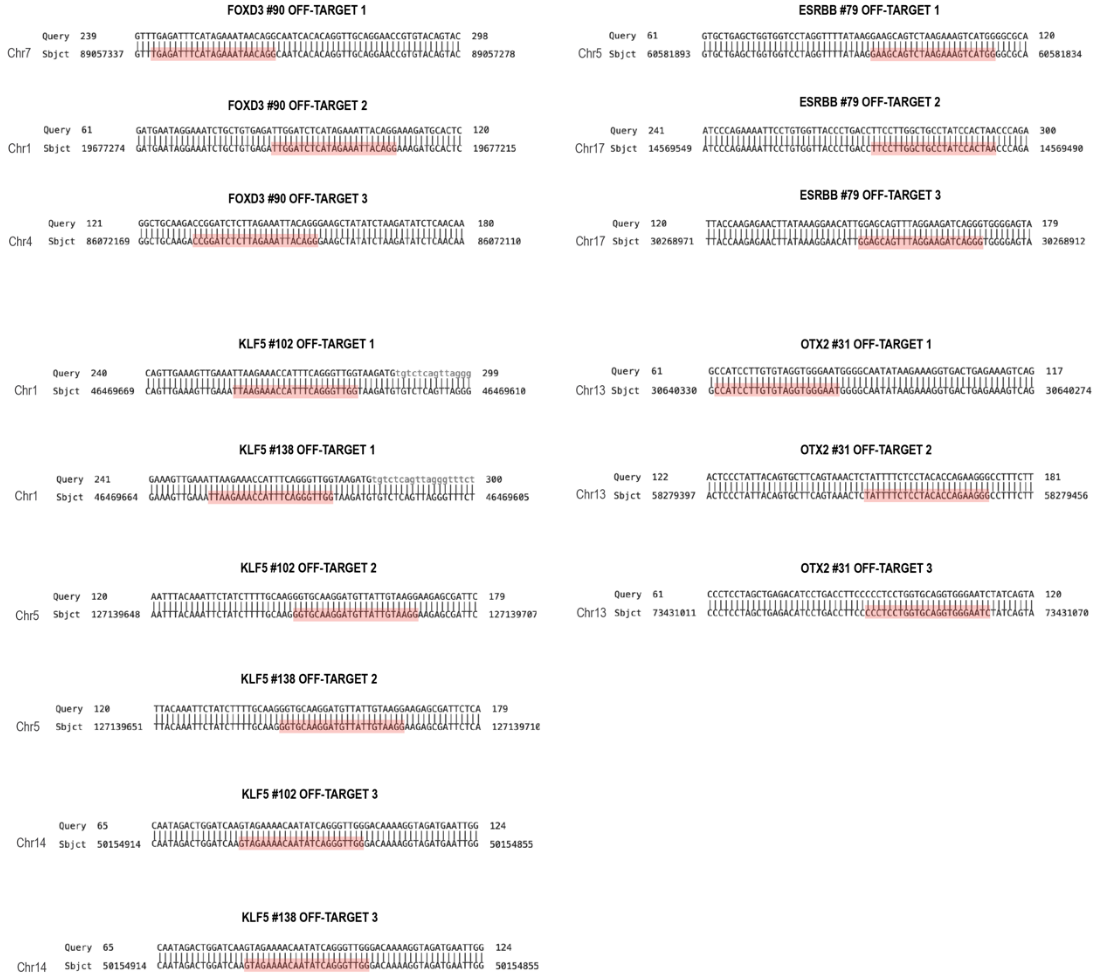

Supplementary Figure 9. OFF-Target analysis in ESC clones with CRISPR-Cas9 deletion of Transcription Factor Binding sites. a, shows the bands containing the OFF-target sequences amplified from each clone's Genomic DNA. See Supplementary Table 4 for primers used. Below the gels, the expected band size is indicated. H lanes are negative controls containing water instead of Genomic DNA solution. **b** shows the alignment of the sequence of the amplified bands with the GRCm39 C57Bl/6J genome sequence. The OFF-target sequences appear highlighted in red.

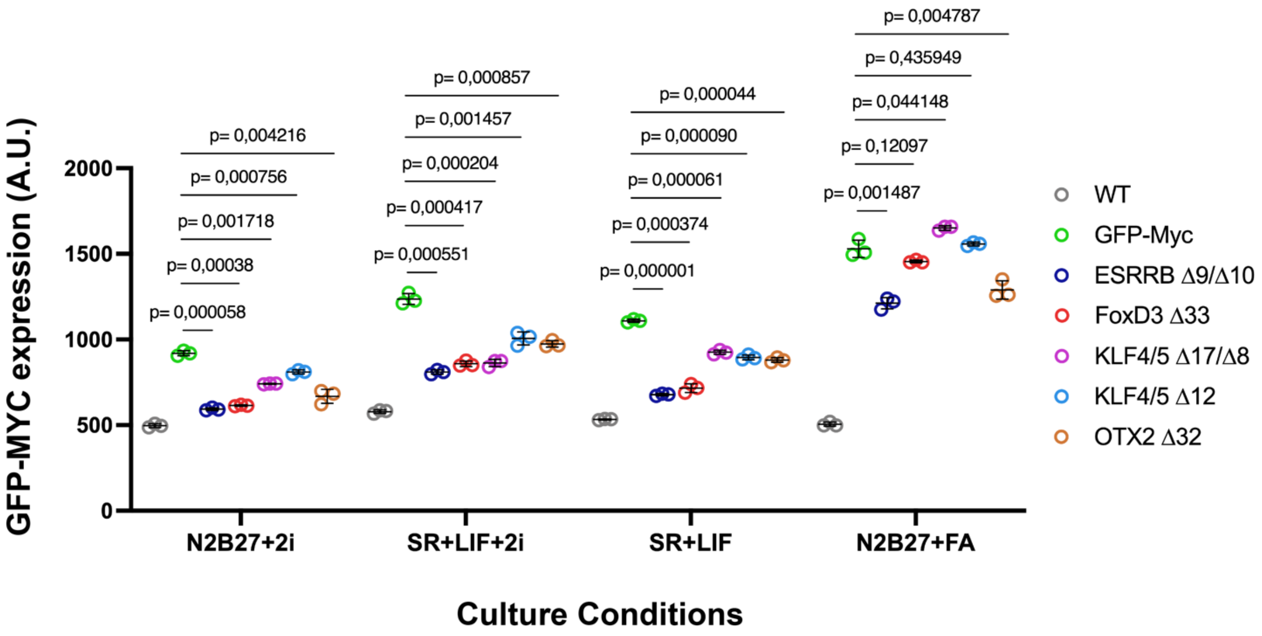

Supplementary Figure 10. GFP-MYC expression levels in mESCs deleted for ESRRB, FoxD3, KLF4/5 and OTX2 candidate binding sites. Dot plot shows flow cytometry GFP-MYC mean intensity of 3 biological replicates of WT, $Myc^{GFP/GFP}$ and $Myc^{GFP/GFP}$ deleted in homozygosity or trans-heterozygosity for the indicated mutations in four culture conditions shown on the X axis. The number of cells quantified per clone/biological replicate is available from the source data in Figshare. Ordinary one-way ANOVA with Šídák's multiple comparisons test. Exact P-values are shown above each comparison. Source data for all graphs are available from the Source Data file and raw data from Figshare (see Data Availability section).

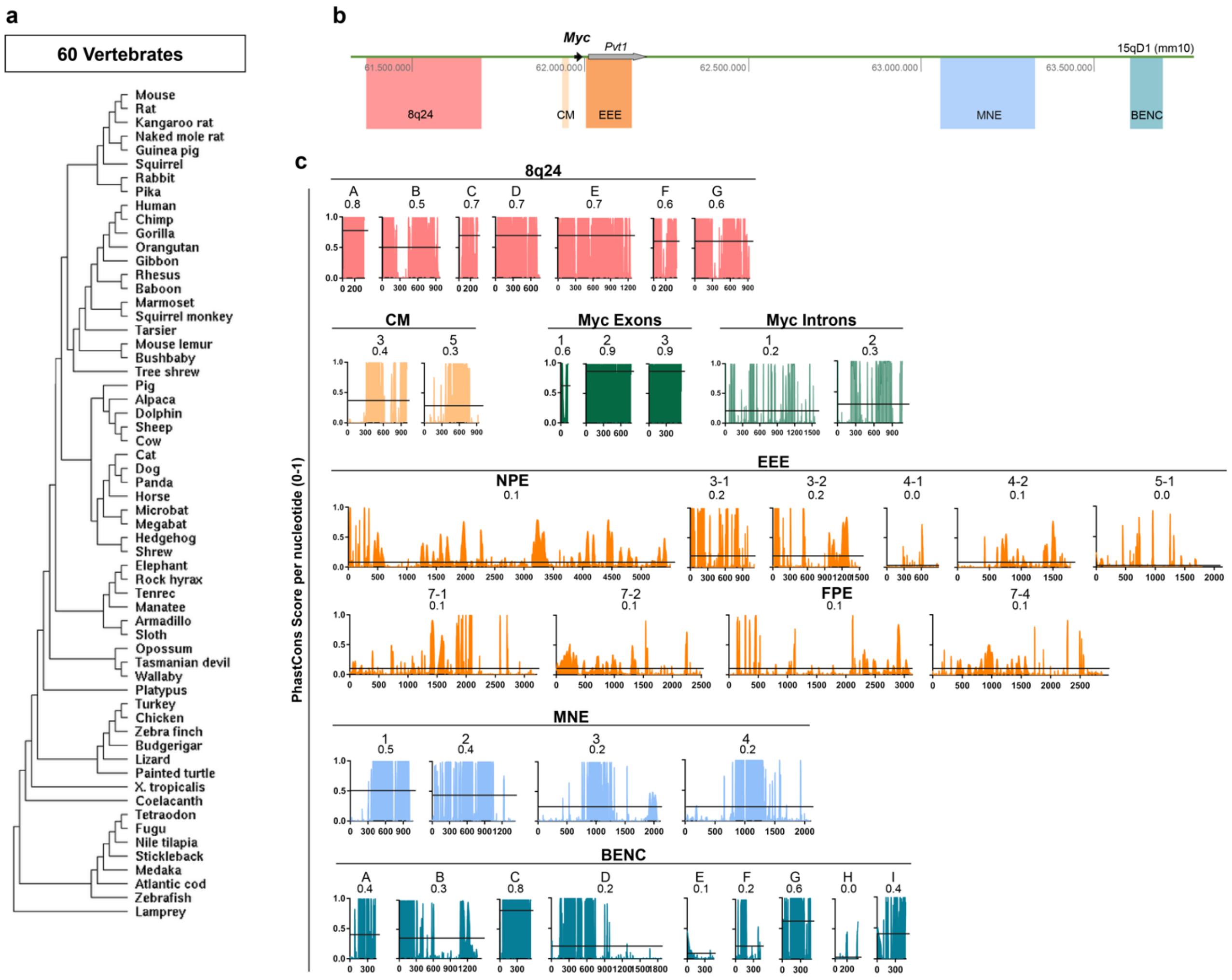

Supplementary Figure 11. Evolutionary conservation of *Myc* enhancers. a, The 60 vertebrate species included in this comparative study. **b,** scaled representation of previously described enhancers belonging to different enhancer cluster regions and the Early Embryonic Expression region described here. Including 8q24 enhancers A-G, CM3, CM5, EEE enhancers 2 to 7-4 (including NPE and FPE), MNE enhancers 1-4 and BENC enhancers A to I. *Myc* (black arrow) and Pvt1 (grey arrow) transcription units are illustrated. The location, size and reference for all the elements shown are listed in Supplementary Table 5. **c,** Graphs show conservations as PhastCons scores (ranged from 0 to 1) per nucleotide. The average of the conservation scores of each element is represented by a black horizontal line and the numerical value appears above the graph.

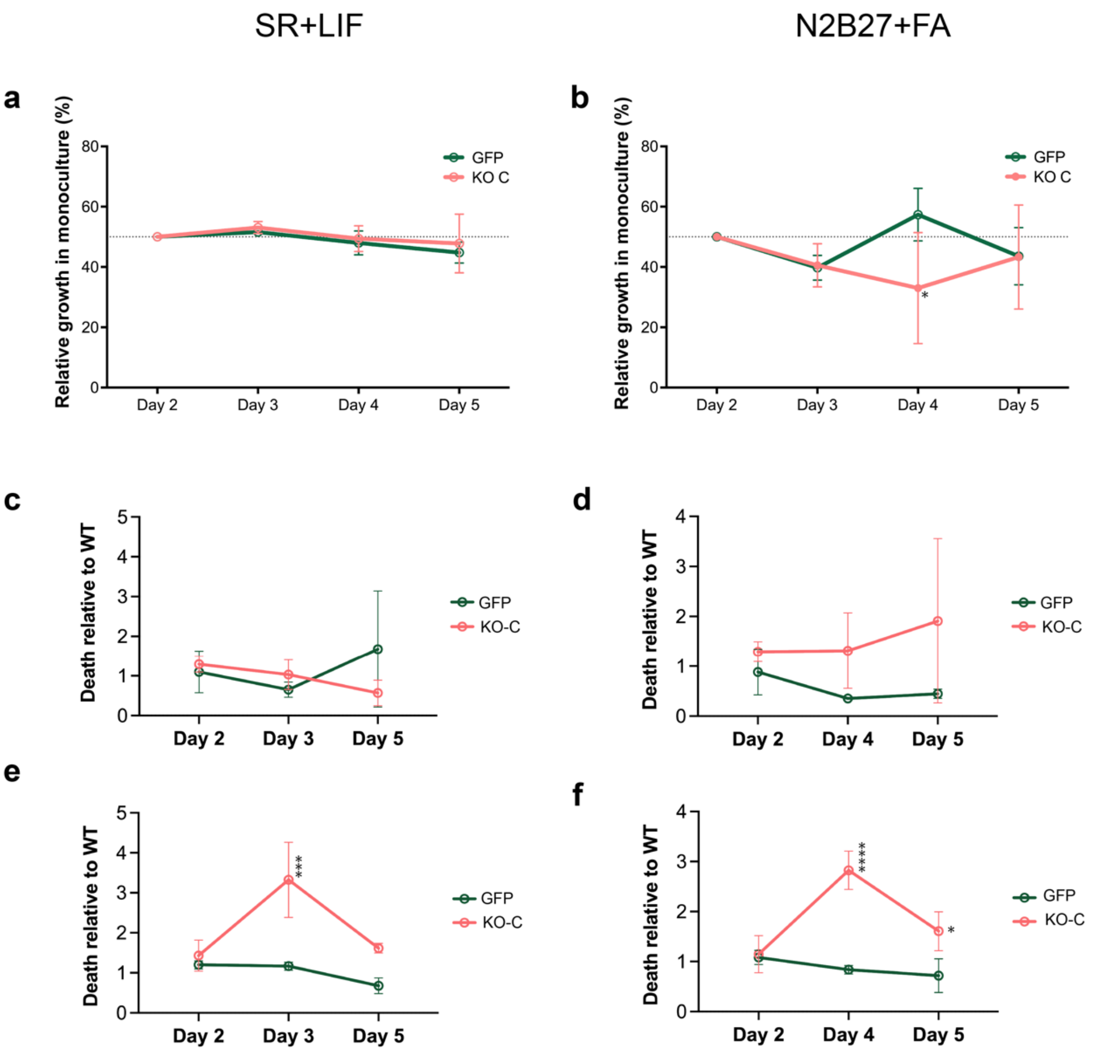

Supplementary Figure 12. Proliferation and cell death in ESCs with Cluster-C deletion.
a, shows the relative proportion between the populations of GFP-Myc ESCs and GFP-Myc ESCs carrying a sub-cluster-C deletion with Control Tomato+ cells cultured independently in SR+LIF. **b** shows similar results when ESCs are cultured in N2B27+F/A conditions. All populations were cultured independently and mixed 1:1 in volume for characterization of the cell populations proportions by Flow Cytometry. **c** and **d** show the proportion of cell death measured in the parental GFP ESC line and in ESCs carrying a sub-cluster-C deletion when cultured independently in SR+LIF (**c**) or N2B27+F/A (**d**). **e** and **f** show similar results, respectively, when cells were co-cultured with the Tomato+ control cells. Cell death was determined by DAPI incorporation in Cytometry. 3 independent clones were measured for each genotype in **a**-**f**. Dots indicate the Mean and error bars show the standard deviation. The number of cells quantified per clone/biological replicate is available from the source data in Figshare. Two-way ANOVA with Šídák's multiple comparisons test; ns: P-value >0,05 (not shown); *: P-value=0,0448 in **b** and 0.0127 in **f**; ***: P-value=0,0001; ****: P-value<0,0001. Source data for all graphs are available from the Source Data file and raw data from Figshare (see Data Availability section).

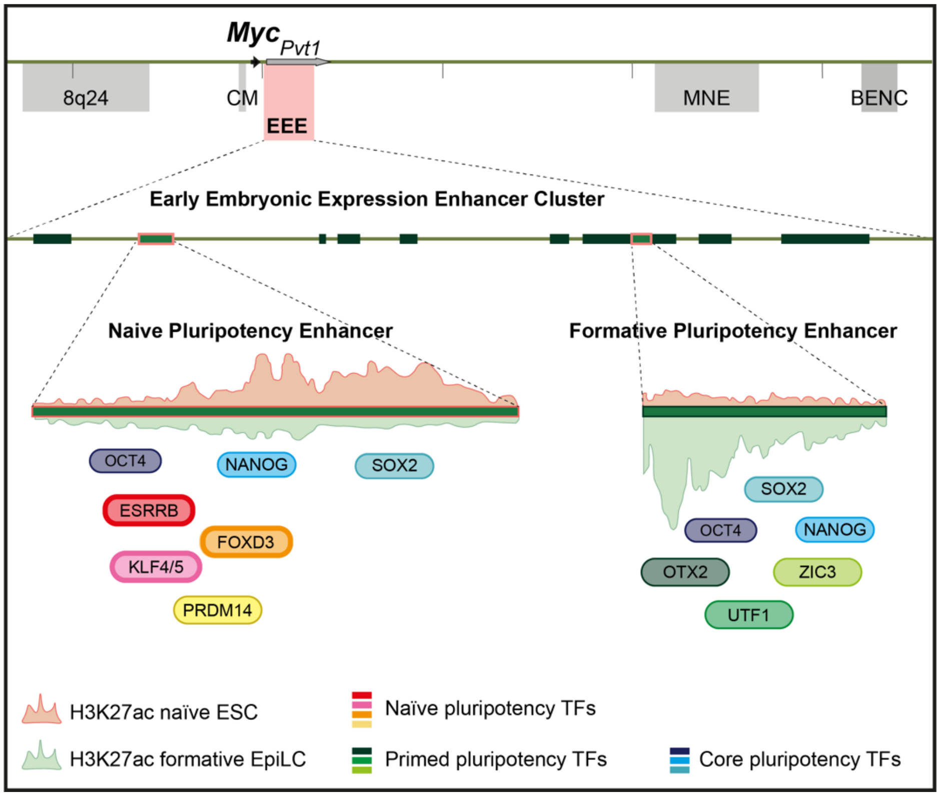

Supplementary Figure 13. Model for the modular regulation of Myc transcription in early mammalian embryos and during different pluripotency states

Supplementary tables

Name	Chr	Localization (mm10)	Type
MycDel3Mb	chr15	chr15:60301761-63718995	Deletion
InvMyc1	chr15	chr15:60301761-62336788	Invertion
Delcent3	chr15	chr15:61518771-61982613	Deletion
Del40	chr15	chr15:62336788-63353788	Deletion
Del11	chr15	chr15:62836993-63353788	Deletion
Del21	chr15	chr15:63364914-63718995	Deletion
**BAC**	**chr15**	**chr15:61958143-62188886**	**RP24-78D24**

Supplementary Table 1. Chromosome rearrangements and BAC localization. mm10 genome coordinates

4C			
		**Forward**	**Reverse**
**PCR1**	adapter	AATGATACGGCGACCACCGAACACTCTTTCCCTAC ACGACGCTCTTCCGATCTtggggcacaagctggagtac-	CAAGCAGAAGACGGCATACGA GTGTCGCCCTCGAACT
**PCR2**	Index-Library	AATGATACGGCGACCACCGAGATCTACACTCTTTC CCTACACGACGCTCTTCCGATCT	(GFP 2I) Rv P7 index1: 5´CAAGCAGAAGACGGCATACGAGATCGTGAT GTGACTGGAGTTCAGACGTGTGCT-3'
			(GFP 2I) Rv P7 index2: 5´- CAAGCAGAAGACGGCATACGAGATACATCGG TGACTGGAGTTCAGACGTGTGCT-3'
			(GFP EPI) Rv P7 index3: 5´- CAAGCAGAAGACGGCATACGAGATGCCTAAG TGACTGGAGTTCAGACGTGTGCT-3'
			(GFP EPI) Rv P7 index4: 5´- CAAGCAGAAGACGGCATACGAGATTGGTCAG TGACTGGAGTTCAGACGTGTGCT-3'
			(TFP 2I) Rv P7 index5: 5´- CAAGCAGAAGACGGCATACGAGATCACTGTG TGACTGGAGTTCAGACGTGTGCT-3'
			(TFP 2I) Rv P7 index6: 5´- CAAGCAGAAGACGGCATACGAGATATTGGCG TGACTGGAGTTCAGACGTGTGCT-3'
			(TFP EPI) Rv P7 index7: 5´- CAAGCAGAAGACGGCATACGAGATGATCTGG TGACTGGAGTTCAGACGTGTGCT-3'
			(TFP EPI) Rv P7 index8: 5´- CAAGCAGAAGACGGCATACGAGATTCAAGTG TGACTGGAGTTCAGACGTGTGCT-3'

Supplementary Table 2. 4C primers. mm10 genome coordinates

crRNAs used for CRISPR deletions				
	**Localization (mm10)**	**Size (bp)**	**5´**	**3´**
**Putative enhancers cluster**				
**Cluster A**	chr15:61,990,505-62,014,068	23.764	GAATGGCTTCGGTTAACTGT	TGTACATATCCGCACCTTCC
			GGGTGTCCTTCCGCTGGTTA	GTTGAACTGAAAGCGACCAA
**Cluster B**	chr15:62,039,218-62,055,983	16.664	GACTGGGAAAAACCTCGTGG	ACCTCTGACAAAGCCTACGA
			GTTGAGTTCAAATAACCGTA	GAAGGAGCTACGTCAACCTA
**Cluster C**	chr15:62,078,497-62,099,928	22.185	CCAGCCTTAAGTGAATGACC	TGCCATTCATGTCGCTGACT
			TCCCAGCAGGGGCACGTGAT	TAAACTGAGCCGGTAACCCG
**Cluster D**	chr15:62,103,836-62,133,033	28.395	GTGGGCTGCGTTCGTTATTC	GTGTACAATGAGCGTAGTCA
			ACTCCCTTGAACACGGGTCC	CTAGGAAGCCTTGTTCGATG
**Putative enhancers**				
**Cluster A-enhancer 2**	chr15:62,008,447-62,014,334	5.888	AGCTGCAGTGATCCAGTACG	TGTACATATCCGCACCTTCC
			CAATGTCCTGTTACGGATTA	GTTGAACTGAAAGCGACCAA
**Cluster C-enhancer 7-1**	chr15:62,085,072-62,088,281	3.211	CTATGTTTGCCTGCGTACCC	TGAGATTGAGATCCGTTCTC
			AGTTCCCTTAACAAGGCCAC	TATCGTAGTTAAGTTTCCCA
**Cluster C-enhancer 7-2**	chr15:62,089,322-62,092,601	3.281	GAGTTGTGACATGCCATACT	CGCATGTGCTATTGCCTTTC
			GTTTAAGGGTGCATCTCGGC	GAGGATTGGATTCCCCTCCT
**Cluster C-enhancer 7-3**	chr15:62,092,596-62,095,870	3.276	GGCCCCTGTGTGTCGATAAC	TTCATTCTAGCATGTGCAGG
			CCAAGGTCAGGTTGTCGGGG	AAGGTATGTCATATCCAAGT
**Cluster C-enhancer 7-4**	chr15:62,096,720-62,099,694	2.976	GACAGTACCTCGCTCAGAAT	AAGCAGTATCTAGAGGACGT
			AAAGAACACATGTAAAGTCG	AAGTGAAGCTTTCTATGACG
**TF binding sites (BS)**				
**ESRRB BS**	chr15:62009970-62009979	9	GAAGCAGTCAAGGAAGGTCA	
	chr15:62009963-62009971	10		
**FoxE1 BS**	chr15:62010890-62010923	33	TTGGATCTCTTAGAAATAAC	
**KLF5 a BS**	chr15:62010999-62011015	17	GTAGGTAACCATTTCAGGGT	
	chr15:62,011,006-62,011,013	8		
**KLF5 b BS**	chr15:62011000-62011011	12		
**OTX2 BS**	chr15:62092929-62092960	32/33	CATTTCCTCCTACACCAGGA	

Supplementary Table 3. crRNA used for sub-cluster and putative enhancer deletions

Primers for genotyping					
	allele	size (pb)	Forward	Reverse	
**Putative enhancers cluster**					
Cluster A	KO	466/1663	TATGGGAAGGGGTTATCATTG	AGTGGTCTTCTTGCCTCTG	
	WT	680	TCCTTTGTACATCAAGCC	GTTGAAGAATCAAAAGTCTG	
Cluster B	KO	568/645	TGTGTGTCTGTGTGTTGG	GCAAGTAGAGCATGCCTG	
	WT	538	CAGTGTTGTTAGCATGTTG	GCAATAGTGGCATGCATG	
Cluster C	KO	444/1136	GAGACAGGGTCTCTAAGTAG	AGATCTAGTTCCAACAATG	
	WT	530	TAGTCTGCCTGTCAAAAC	AAGGACAGCAGTAAACTAAG	
Cluster D	KO	451/751	TCTGGCTTATTGGGTCCCTG	CCAGGAACAGTTTTTGAAAC	
	WT	398	GCTCTGCCTCTCAGCTCAC	CACACCCACACATCTCTG	
**Putative enhancers**					
Cluster A-enhancer 2	KO	952	GTAAGGAGGTTGAAATGGCTCAAG	GGGAGTAATGGATGCTGTGGT	
	WT	1496	CTTCTCCTGGTTGACACGCT	ATTGTGGTTTTCTGCCGTGC	
Cluster C-enhancer 7-1	KO	585/855	TGGTATGGACAGCATTGTAG	CTATACCTAGTACTCGGAG	
	WT	524	GCTGTTGCTTGCTCTGGC	AGCTGGGCATTAACACCAC	
Cluster C-enhancer 7-2	KO	506/700	TCCTGGGCCTGAAATAAC	GCCTAGGCAGCATCTGGC	
	WT	500	TCTCGAATCTTTGAGATGTTTC	TACTCTGGGGAGGAGATATTC	
Cluster C-enhancer 7-3	KO	388/401	CACCTTTATGAGAGGCCTTAGG	CCCTGTCTTGAAAAACAG	
	WT	505	AGCGTTGCTGTATATCAGATAC	GTCCCAGCACTGAAGAGG	
Cluster C-enhancer 7-4	KO	600/775	CAGATTATCACACCCCAAG	CTGTGGCCAGCAGAGATG	
	WT	541	CTTCTCACCATATGTAAGGGATC	TCTCCTCTGGTGGCAAAAC	
**TF binding sites (BS)**					**PCR products restriction**
**ESRRB BS**	WT/KO	314	CAGGCTGTTTGGAGAGCTGA	TAAAGGGCCACACAGCTGAC	ESRBB_Hinfl (wt): 185+130 bp
					ESRBB_Hinfl (del): 300 bp
**FoxE1 BS**	WT/KO	577	TCTCATCCCTCGCCAAAGA	CAACCCTTCCCATGTCCAG	Foxd3-72_ BstYI (wt): 359 +218 bp
					Foxd3-72_ BstYI (del): 577bp
**KLF5 a BS**	WT/KO	640	TCAGAGACTCTACTGCCCCAT	TCATGTGCTATGTCCTCAGCC	Klf5_BslI (wt): 391 +125 + 63 +61 bp
**KLF5 b BS**					Klf5_BslI (del): 454 +125 +61 bp
**OTX2 BS**	WT/KO	335	TAAGGAGGGGAGGAAGAGGC	CTGGAAGGAAAGGCAGGGAG	Otx2_BstNI (wt): 118 + 76 + 60+ 57+ 24pb
					Otx2_BstNI (del): 133 + 118+ 60+ 24 pb

OFF-Target Sequence amplification					OFF-target position	MIT OFF-Target Score	OFF-target Sequence
ESRBB crRNA	1	428	CTTCTGAAAGGAGGAGTCATCAA	TTGGAACTCTATTGCTGGGATAA	chr5:60424497-60424519:-	0.20	GAAGCAGTCTAAGAAAGTCATGG
	2	427	TGTCCTGCTTGCTTCCTCTG	GGCCATTCCAGGTAAGCCTTC	chr17:14349242-14349264:+	2.51	TAGGCAGCCAAGGAAGGTCAGGG
	3	476	TGTGCATAATAGTCCCTTTACCG	ATACGTTTGAGTGCTTGGACTTC	chr19:30291521-30291543:-	0.34	GGAGCAGTTTAGGAAGATCAGGG
KLF5 crRNA	1	600	TGATTTGCCTTTAGTGGATAGCT	TCTCTGAGGACTGGTCTGGAAG	chr1:53702359-53702381:+	1.47	TTAAGAAACCATTTCAGGGTTGG
	2	440	TAAGCTAAGCAATCTCACCAGC	GAGTTTCAAAAGAGTCCTCCAGG	chr5:127062610-127062632:+	0.37	GGTGCAAGGATGTTATTGTAAGG
	3	465	CCCGAACTTACTAATCTCCCAGT	CACAATAGAGGAGTGGTTGGGT	chr14:49917419-49917441:-	0.29	GTAGAAAACAATATCAGGGTTGG
FoxE1 crRNA	1	535	CCTGCATCACTTCCTTCACA	TTGTGCTATGTTGGGAACCA	chr7:89408104-89408126:-	0.85	TGAGATTTCATAGAAATAACAGG
	2	492	GAAAACCAAAACCCATCCAA	AATTCCCCCTTTCCTTTCCT	chr1:19607003-19607025:-	1.36	TTGGATCTCATAGAAATTACAGG
	3	473	GTTCTTTCACCCCTCAAACAAG	GGCTCATCTCTGGGAAAGACTA	chr4:86153900-86153922:-	0.66	CCGGATCTCTTAGAAATTACAGG
OTX2 crRNA	1	510	ACATAATTGAGGGAAGCCAGGG	AAGGGACCAAGCATGACTGAAT	chr13:30456324-30456346:+	0.48	CATTCCCACCTACACAAGGATGG
	2	532	TGAGGGTAGGAACCCGAGAG	CTCTCACTACCCCCACTCCA	chr13:58131612-58131634:+	0.68	TATTTTCTCCTACACCAGAAGGG
	3	485	TGAGAGGCAGGGTGCTATCT	ATCCACCAGAGAGAGGCCATT	chr13:73282921-73282943:-	0.70	CCCTCCTGGTGCAGGTGGGAATC

Supplementary Table 4. Primers for genotyping CRISPR-Cas9 deletions and OFF-target sites. mm10 genome coordinates

name	ref	coordinates (mm10)	size bp
8q24 enhancer A	Sotelo et al., 2010	chr15:61356836-61357193	358
8q24 enhancer B	Sotelo et al., 2010	chr15:61365625-61366591	967
8q24 enhancer C	Sotelo et al., 2010	chr15:61395374-61395696	323
8q24 enhancer D	Sotelo et al., 2010	chr15:61608209-61608962	754
8q24 enhancer E	Sotelo et al., 2010	chr15:61618832-61620086	1.255
8q24 enhancer F	Sotelo et al., 2010	chr15:61653313-61653698	386
8q24 enhancer G	Sotelo et al., 2010	chr15:61689984-61690912	929
CM3	Hallikas et al., 2006	chr15:61949429-61950423	995
CM5	Hallikas et al., 2006	chr15:61959148-61960079	932
Myc	exon (UCSC)	chr15:61,985,922-61,989,908	1369
	exon 1	chr15:61,985,922-61,985,951	30
	exon 2	chr15:61,987,504-61,988,279	776
	exon 3	chr15:61,989,346-61,989,908	563
Myc	intron (UCSC)	chr15:61,985,922-61,989,908	2.622
	intron 1	chr15:61,985,951-61,987,504	1.554
	intron 2	chr15:61,988,279-61,989,346	1.068
Enhancer 2	ROI	chr15:62,008,447-62,014,334	5.888
Cluster B-enhancer 3-1	ROI	chr15:62,038,009-62,039,109	1.101
Cluster B-enhancer 3-2	ROI	chr15:62,039,143-62,040,653	1.511
Cluster B-enhancer 4-1	ROI	chr15:62,043,253-62,044,145	893
Cluster B-enhancer 4-2	ROI	chr15:62,044,235-62,046,157	1.923
ClusterB-enhancer 5-1	ROI	chr15:62,052,822-62,054,955	2.134
Cluster C-enhancer 7-1	ROI	chr15:62,085,072-62,088,281	3.210
Cluster C-enhancer 7-2	ROI	chr15:62,089,322-62,092,601	3.280
Cluster C-enhancer 7-3	ROI	chr15:62,092,596-62,095,870	3.275
Cluster C-enhancer 7-4	ROI	chr15:62,096,720-62,099,694	2.975
MNE Enhancer 1	Uslu et al., 2014	chr15:63052149-63053208	1.060
MNE Enhancer 2	Uslu et al., 2014	chr15:63163294-63164714	1.421
MNE Enhancer 3	Uslu et al., 2014	chr15:63189194-63191298	2.105
MNE Enhancer 4	Uslu et al., 2014	chr15:63339415-63341511	2.097
BENC-A	Bahr et al., 2018	chr15:63611116-63611556	441
BENC-B	Bahr et al., 2018	chr15:63616791-63618232	1.442
BENC-C	Bahr et al., 2018	chr15:63648126-63648667	542
BENC-D	Bahr et al., 2018	chr15:63657837-63659699	1.863
BENC-E	Bahr et al., 2018	chr15:63665359-63665959	601
BENC-F	Bahr et al., 2018	chr15:63678942-63679359	418
BENC-G	Bahr et al., 2018	chr15:63704706-63705184	479
BENC-H	Bahr et al., 2018	chr15:63709542-63709926	385
BENC-I	Bahr et al., 2018	chr15:63736276-63736746	471

Supplementary Table 5. Location, reference and size of the enhancers used for the conservation study. mm10 genome coordinates

Uncropped gels from Supplementary Figure 4

a

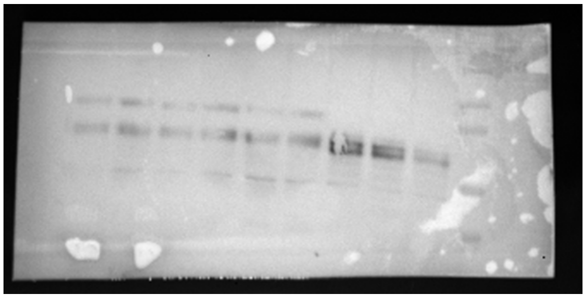

b

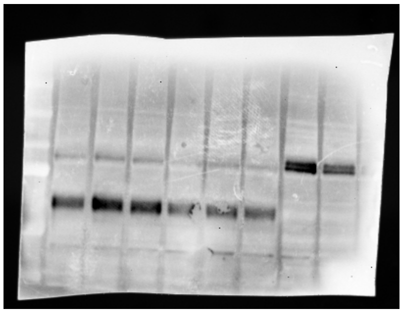

c

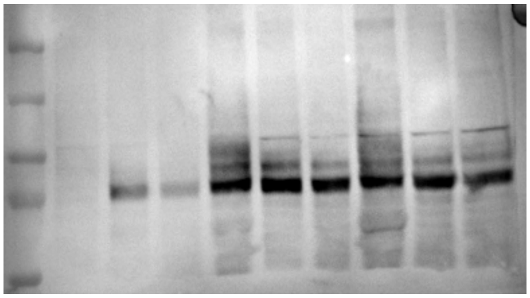

d

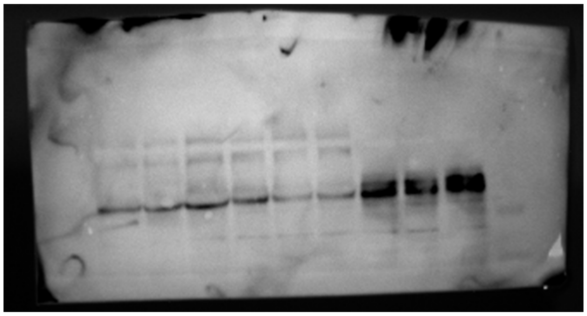

e

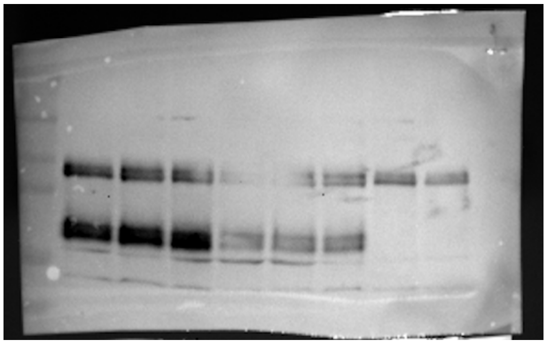

Supplement: Supplementary file 1 — Supplementary Information [file 41467_2024_48258_MOESM1_ESM.pdf]
