# Supplementary material for: Regulation of Myc transcription by an enhancer cluster dedicated to pluripotency and early embryonic expression
Source: Nat Commun. 2024 May 10;15:3931. doi: 10.1038/s41467-024-48258-5 (PMC11087473; doi:10.1038/s41467-024-48258-5)
Supplement: Supplementary file 3 — Reporting Summary [file 41467_2024_48258_MOESM3_ESM.pdf]

nature portfolio

Corresponding author(s): Miguel Torres

Last updated by author(s): Mar 21, 2024

Reporting Summary

Nature Portfolio wishes to improve the reproducibility of the work that we publish. This form provides structure for consistency and transparency in reporting. For further information on Nature Portfolio policies, see our Editorial Policies and the Editorial Policy Checklist.

Statistics

For all statistical analyses, confirm that the following items are present in the figure legend, table legend, main text, or Methods section.

n/a	Confirmed	
☐	☒	The exact sample size (*n*) for each experimental group/condition, given as a discrete number and unit of measurement
☐	☒	A statement on whether measurements were taken from distinct samples or whether the same sample was measured repeatedly
☐	☒	The statistical test(s) used AND whether they are one- or two-sided<br>*Only common tests should be described solely by name; describe more complex techniques in the Methods section.*
☐	☒	A description of all covariates tested
☐	☒	A description of any assumptions or corrections, such as tests of normality and adjustment for multiple comparisons
☐	☒	A full description of the statistical parameters including central tendency (e.g. means) or other basic estimates (e.g. regression coefficient) AND variation (e.g. standard deviation) or associated estimates of uncertainty (e.g. confidence intervals)
☐	☒	For null hypothesis testing, the test statistic (e.g. *F*, *t*, *r*) with confidence intervals, effect sizes, degrees of freedom and *P* value noted<br>*Give P values as exact values whenever suitable.*
☒	☐	For Bayesian analysis, information on the choice of priors and Markov chain Monte Carlo settings
☒	☐	For hierarchical and complex designs, identification of the appropriate level for tests and full reporting of outcomes
☒	☐	Estimates of effect sizes (e.g. Cohen's *d*, Pearson's *r*), indicating how they were calculated

Our web collection on statistics for biologists contains articles on many of the points above.

Software and code

Policy information about availability of computer code

Data collection	FloJo_v10_8_1 ; Leica bulit-in acquisition software for TCS SP8 Navigator System ; Cyto¬vision v 7.4.0.0
Data analysis	ImageJ2 version 2.14.0/1.54f ; GraphPad Prism 5.0a ; Cellpose 2.0 ; Imaris 8 ; CNVnator (https://github.com/abyzovlab/CNVnator) ; pipe4C (https://github.com/deLaatLab/pipe4C)

For manuscripts utilizing custom algorithms or software that are central to the research but not yet described in published literature, software must be made available to editors and reviewers. We strongly encourage code deposition in a community repository (e.g. GitHub). See the Nature Portfolio guidelines for submitting code & software for further information.

Data

Policy information about availability of data

All manuscripts must include a data availability statement. This statement should provide the following information, where applicable:

- Accession codes, unique identifiers, or web links for publicly available datasets
- A description of any restrictions on data availability
- For clinical datasets or third party data, please ensure that the statement adheres to our policy

The Source data generated in this study have been deposited in the Figshare database and are available from https://doi.org/10.6084/m9.figshare.24794559. The data for some images that were not quantified but only qualitatively analysed and were too large for the allowance in the Figshare repository can be obtained from M.T. upon request. The sequencing data generated in this study are available from the GEO database (https://www.ncbi.nlm.nih.gov/geo/) with accession number

GSE222299. The ChIPseq data used in this study are publicly available from the ChIPseq Atlas (https://chip-atlas.org/peak) and the PlacSeq data used in this study are available from the 3Dgenome database (http://3dgenome.fsm.northwestern.edu/).

Research involving human participants, their data, or biological material

Policy information about studies with human participants or human data. See also policy information about sex, gender (identity/presentation), and sexual orientation and race, ethnicity and racism.

Reporting on sex and gender	N/A
Reporting on race, ethnicity, or other socially relevant groupings	N/A
Population characteristics	N/A
Recruitment	N/A
Ethics oversight	N/A

Note that full information on the approval of the study protocol must also be provided in the manuscript.

Field-specific reporting

Please select the one below that is the best fit for your research. If you are not sure, read the appropriate sections before making your selection.

☒ Life sciences ☐ Behavioural & social sciences ☐ Ecological, evolutionary & environmental sciences

For a reference copy of the document with all sections, see nature.com/documents/nr-reporting-summary-flat.pdf

Life sciences study design

All studies must disclose on these points even when the disclosure is negative.

Sample size	No sample size was calculated
Data exclusions	No data were excluded
Replication	Biological replicates were used when indicated and results were compatible between replicates in all cases
Randomization	No randomization was used. This is a genetic study and allocation of specimens/samples was according to genotype
Blinding	No blinding was used. the image analysis methods used are intrinsically objective (cytometry or automated FIJI plugins that measure fluorescence intensity cell by cell)

Reporting for specific materials, systems and methods

We require information from authors about some types of materials, experimental systems and methods used in many studies. Here, indicate whether each material, system or method listed is relevant to your study. If you are not sure if a list item applies to your research, read the appropriate section before selecting a response.

Materials & experimental systems

n/a	Involved in the study	
☐	☒	Antibodies
☐	☒	Eukaryotic cell lines
☒	☐	Palaeontology and archaeology
☐	☒	Animals and other organisms
☒	☐	Clinical data
☒	☐	Dual use research of concern
☒	☐	Plants

Methods

n/a	Involved in the study	
☒	☐	ChIP-seq
☐	☒	Flow cytometry
☒	☐	MRI-based neuroimaging

Antibodies

Antibodies used	Antibodies for Western Blot: MYC (rabbit, Abcam, #ab32072), GFP (rabbit, Takara, #632593) and Vinculin (mouse, Merk,

Antibodies used	#V4505-100UL) were used at 1:1000 . Anti-rabbit-HRP (Dako, #0448) and anti-mouse-HRP (Dako, #0447) were used at 1:500 Antibodies for Whole-Mount Immunofluorescence:GFP Goat Polyclonal Antibody (Goat, 1:200, OriGene, #R1091AP), Human/Mouse Brachyury Antibody (mouse, 1:250, R&D systems, #AF2085-SP) and Anti-SOX2 antibody (rabbit, 1:200, Abcam, #ab97959). Secondary antibodies: 648 donkey anti-goat (1:500, Life Tech, #A-21447), 488 goat anti-mouse (1:500, Life Tech, #A32723) and 633 goat anti-rabbit (1:500, Life Tech, #A-21070). Antibodies for immunofluorescence in cultured cells: Primary antibodies were: phospho-p44/42 MAPK (Erk1/2) (Thr202/Tyr204) (rabbit, 1:200, Cell Signaling, #4370 #9106), Anti-SOX2 antibody (rabbit, 1:200, Abcam, #ab97959) and Oct-3/4 Antibody (C-10) (mouse, 1:200, Santa Cruz, #sc-5279). Secondary antibodies: 633 goat anti-rabbit (1:500, Life Tech, #A-21070) and 568 goat anti-mouse (1:500, Life Tech, #A-11004).
Validation	All antibodies displayed distribution patterns compatible with previously described signals in mouse embryos and mouse embryonic stem cells

Eukaryotic cell lines

Policy information about cell lines and Sex and Gender in Research

Cell line source(s)	Mouse male Embryonic Stem Cells derived from pre-implantation mouse embryos
Authentication	Mutant cells were generated and genotyped in-house and are described in the manuscript for the first time. R, G4 and C57BL/6 wild type ES cells were received from the laboratories that generated the cells and were tested for in vitro properties and markers and for the ability to contribute to the mouse germline when reintroduced in pre-implantation embryos
Mycoplasma contamination	All lines are periodically tested for Mycoplasma and found negative
Commonly misidentified lines (See ICLAC register)	N/A

Animals and other research organisms

Policy information about studies involving animals; ARRIVE guidelines recommended for reporting animal research, and Sex and Gender in Research

Laboratory animals	GFP-MYC mice on a C57BL/6JCrl background were described in doi:10.1002/eji.200737972 and obtained from the creator laboratory. TFP-Myc transgenic mice were generated in-house by standard transgenesis methods on a C57BL/6JCrl background. Wild type mice of the CD1 strain were bred in house and used for experiments not involving genetically modified mice. All specimens analyzed were early embryos and therefore sex is not relevant at this stage.
Wild animals	N/A
Reporting on sex	All specimens analyzed were early embryos before gastrulation and therefore sex was not determined and is not relevant at this stage of development.
Field-collected samples	N/A
Ethics oversight	Animals were handled in accordance with CNIC Ethics Committee, Spanish laws and the EU Directive 2010/63/EU for the use of animals in research. All mouse experiments were approved by the Centro Nacional de Investigaciones Cardiovasculares and Universidad Autónoma de Madrid Committees for “Ética y Bienestar Animal” and the area of “Protección Animal” of the Community of Madrid with references PROEX 220/15 and PROEX 144.1/21.

Note that full information on the approval of the study protocol must also be provided in the manuscript.

Plants

Seed stocks	*Report on the source of all seed stocks or other plant material used. If applicable, state the seed stock centre and catalogue number. If plant specimens were collected from the field, describe the collection location, date and sampling procedures.*
Novel plant genotypes	*Describe the methods by which all novel plant genotypes were produced. This includes those generated by transgenic approaches, gene editing, chemical/radiation-based mutagenesis and hybridization. For transgenic lines, describe the transformation method, the number of independent lines analyzed and the generation upon which experiments were performed. For gene-edited lines, describe the editor used, the endogenous sequence targeted for editing, the targeting guide RNA sequence (if applicable) and how the editor was applied.*
Authentication	*Describe any authentication procedures for each seed stock used or novel genotype generated. Describe any experiments used to assess the effect of a mutation and, where applicable, how potential secondary effects (e.g. second site T-DNA insertions, mosiacism, off-target gene editing) were examined.*

Flow Cytometry

Plots

Confirm that:

☒ The axis labels state the marker and fluorochrome used (e.g. CD4-FITC).

☒ The axis scales are clearly visible. Include numbers along axes only for bottom left plot of group (a 'group' is an analysis of identical markers).

☒ All plots are contour plots with outliers or pseudocolor plots.

☒ A numerical value for number of cells or percentage (with statistics) is provided.

Methodology

Sample preparation	cells were trypsinized, stained for DAPI and analyzed for endogenous GFP or TFP signal and DAPI
Instrument	BD FACSAriaTM II
Software	Flowjo
Cell population abundance	N/A. No sorting was used
Gating strategy	Gating was done on the DAPI channel selecting individual healthy cells

☒ Tick this box to confirm that a figure exemplifying the gating strategy is provided in the Supplementary Information.
